# Single trial decoding of belief decision making from EEG and fMRI data using independent components features

**DOI:** 10.3389/fnhum.2013.00392

**Published:** 2013-07-31

**Authors:** Pamela K. Douglas, Edward Lau, Ariana Anderson, Austin Head, Wesley Kerr, Margalit Wollner, Daniel Moyer, Wei Li, Mike Durnhofer, Jennifer Bramen, Mark S. Cohen

**Affiliations:** ^1^LINT Laboratory, University of California, Los AngelesLos Angeles, CA, USA; ^2^Department of Neurology, University of California, Los AngelesLos Angeles, CA, USA; ^3^Interdepartmental Program in Neuroscience, University of California, Los AngelesLos Angeles, CA, USA; ^4^California Nanosystems Institute, University of California, Los AngelesLos Angeles, CA, USA

**Keywords:** machine learning, decoding, EEG, fMRI, ICA, decision making, decision tree, interpretation

## Abstract

The complex task of assessing the veracity of a statement is thought to activate uniquely distributed brain regions based on whether a subject believes or disbelieves a given assertion. In the current work, we present parallel machine learning methods for predicting a subject's decision response to a given propositional statement based on independent component (IC) features derived from EEG and fMRI data. Our results demonstrate that IC features outperformed features derived from event related spectral perturbations derived from any single spectral band, yet were similar to accuracy across all spectral bands combined. We compared our diagnostic IC spatial maps with our conventional general linear model (GLM) results, and found that informative ICs had significant spatial overlap with our GLM results, yet also revealed unique regions like amygdala that were not statistically significant in GLM analyses. Overall, these results suggest that ICs may yield a parsimonious feature set that can be used along with a decision tree structure for interpretation of features used in classifying complex cognitive processes such as belief and disbelief across both fMRI and EEG neuroimaging modalities.

## Introduction

The complex process of decision-making appears to engage distinct cortical regions whose spatio-temporal evolution occurs over multiple stages of directed processing. While this processing likely varies according to the specific task and its difficulty, its framework is thought proceed by internal representation of variables, valuation of these internal states, and eventual action selection (Rangel et al., 2008). EEG and fMRI have each been used according to their individual strengths in temporal and spatial precision to measure both serial and parallel aspects of neural computation involved in decision-making in humans (Heekeren et al., 2008).

Neuroimaging studies using fMRI have demonstrated that decision tasks involving perceptual stimuli discrimination consistently activate fronto-parietal networks (White et al., 2012) including the dorsolateral prefrontal cortex (dlPFC). Similar functional activation patterns also emerge in humans during the process of consciously assessing the truth content of a statement, as revealed by fMRI (Harris et al., 2008). Nonetheless, the specific brain loci and patterns of activation appear to vary uniquely according to both the eventual decision outcome, and the categorical decision being made (Heekeren et al., 2003).

Machine learning (ML) methods are now commonly applied to neuroimaging data and have been used predicatively to decode decision responses based on blood oxygenation level dependent (BOLD) signals in selected brain regions (Calvert and Brammer, 2012). However, the volume of data in fMRI is vast—far beyond what can be interpreted readily from a simple localization perspective, and a more parsimonious representation of the data can ease the interpretation process. When applied in a “transparent” fashion, ML methods can also be leveraged for their explanatory power to gain insight into the underpinnings of neural circuitry (O'Toole et al., 2007; Ecker et al., 2010; Hanke et al., 2010).

On the one hand, whole brain voxel data has been used effectively for fMRI decoding (e.g., LaConte et al., 2007), particularly when a classifier such as a support vector machine (SVM) is well tuned on these data (Chu et al., 2012). However, with too many inputs, a classifier may begin to fit the noise, and this overfitting may lead to poor generalization capability (Yamashita et al., 2008). Physiologically-driven approaches such as selecting functional regions of interest (ROIs) diminish input size substantially (Cox and Savoy, 2003; Chu et al., 2012), but require *a priori* knowledge of brain morphology associated with a given task (Mourão-Miranda et al., 2006). While tools like multivoxel pattern analysis (Norman et al., 2006) provide methods for determining voxel subsets with high signal-to-noise ratio, these subsets may differ across individual scans, and spatially adjacent voxels may provide redundant information.

The challenge or extracting class specific signal features from EEG data is similarly challenging. EEG are inherently noisy and non-stationary, varying significantly from trial-to-trial (Müller et al., 2008). Nonetheless, decoding brain states at the single trial level has been made possible by developing analysis tools that explain the high dimensional data with a well-defined underlying structure. While event related potential features derived from the EEG signal itself (e.g., P300) have been used to drive ML based brain computer interfaces (Krusienski et al., 2008), it is often useful to apply a dimension reduction technique first. Common spatial patterns (e.g., Dornhege et al., 2006) that seek to find filters that maximize variance in one condition, principle component analysis (Subasi and Ismail Gursoy, 2010) and analytic signal reconstruction of event related spectral perturbations (D'Zmura et al., 2009) have all been useful for feature extraction in decoding EEG signals. Ideally, the dimension reduction step is both interpretable and capable of being used in the absence of a class specific signature hypothesis (Lal et al., 2004), particularly if the goal is identification of novel EEG components related to a cognitive process.

Using a method such as independent component analysis (ICA) allows basis images to cover the entire brain, and is an *unsupervised* blind source separation technique (Bell and Sejnowski, 1995; Calhoun and Adali, 2006) that does not require a priori physiologic knowledge about a certain brain process. ICA has found numerous applications in fMRI and EEG (Lan et al., 2005) to include: data exploration (Beckmann et al., 2006), noise component elimination (Tohka et al., 2008), and as a basis for decoding analysis (De Martino et al., 2007; Anderson et al., 2009; Douglas et al., 2011). A key advantage is that ICs are nominated by the data themselves. Furthermore, IC spatio-temporal signatures across individuals appear both stable and consistent within functional neural subsystems (Damoiseaux et al., 2006; Smith et al., 2012).

In the present paper, we describe an ICA based ML approach to classify fMRI and EEG data of persons engaged in a bivariate task, asserting their belief or disbelief of a variety of propositional statements. We extend previous work (Douglas et al., 2011) by developing a quantitative metric for comparing IC features with traditional general linear model (GLM) analysis results for interpretation purposes. We then create a parallel ML approach for single trial classification of belief versus disbelief using high-density electrode EEG data, and compare the classification accuracy achieved using ICs derived from each functional modality.

## Methods

### Overview

**Figure 1:**
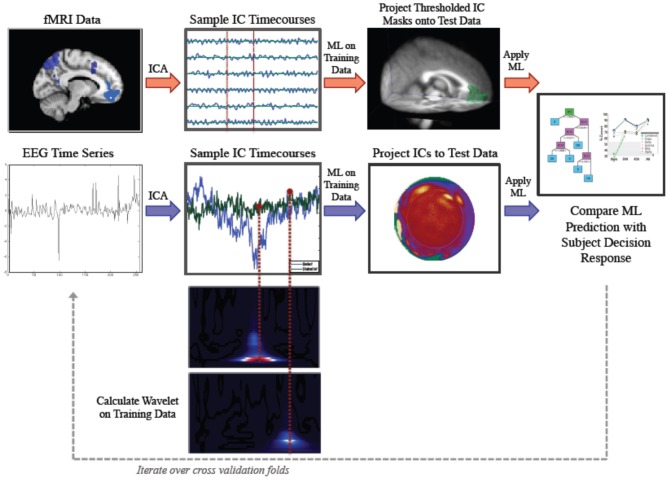
**Parallel Method for Independent Component Analysis (ICA) based discrimination of belief and disbelief using machine learning techniques**. Following ICA decomposition on data exemplars randomly parsed into the training set, FMRI ICs (top, red arrows) are thresholded and binarized. These spatial masks are then multiplied by testing data. Mean activation values are then extracted for each IC, and sampled timecourses are used as features for decoding. EEG IC activations (bottom, blue arrows) are projected onto testing data, and IC activation timecourses are sampled at time points determined by wavelet data, and used as inputs for classification.

Our method involved application of parallel IC processing to both EEG and fMRI data for the purpose of classification of belief decision making. In brief, we collected EEG and fMRI from subjects who were prompted to decide whether they believed or disbelieved a particular statement presented to them on a screen. Decision responses were recorded and used for training and testing a ML classifier. ICA was run on training sets for both fMRI and EEG data. ICs were sampled at time points that were determined to be informative for discrimination. We then projected our ICs forward onto test data and applied our ML classifier to test data. We then calculated accuracy by comparing the subject's keypad response to our ML predicted response. A schematic illustrating the parallel ICA ML processing pipelines for fMRI and EEG is shown in Figure 1.

### Subjects

A total of 37 healthy participants volunteered for this experiment. Written informed consent was obtained from each participant prior to the experiment, which was approved by the UCLA Institutional Review Board. Fourteen subjects participated in the fMRI portion of the study, while 23 participants participated in the EEG portion of the experiment. All subjects were healthy volunteers aged 18–45 years old, with 15 of the participants being female.

### Experimental design

During the experiment, subjects were asked to evaluate truth content from a given statement, and indicate their assessment with a keypad response. Statements were chosen at random from the following categories: mathematical, geographical, semantic factual, autobiographical, religious, and ethical. For the fMRI task, statements were presented via MR-compatible goggles. For additional details about the fMRI stimulus paradigm, and categorical statements see (Harris et al., 2008). For the purposes of our ML analysis here, we collapse all belief and disbelief events across statement category.

The stimuli task paradigm was implemented in MATLAB (Mathworks, Inc.) using the Psychophysics Toolbox, Version 3.0 (Brainard, 1997). Each subject trial began with a brief instructional statement following by a crosshair fixation. Statements were presented in random order as black text against a gray background. For the subjects who participated in the EEG portion of the experiment, a subset of subjects (*n* = 10) viewed each statement using a rapid serial visual presentation (RSVP) protocol with inter-word and inter-stimulus intervals of 500 ms. However, in the present work, we focus on EEG data from subjects that viewed the statements using the same protocol as for the fMRI portion of the experiment. In this design, the entire statement was presented on the screen at once. Each statement was centered on the screen with new lines beginning after each set of four words to minimize saccade artifact. Progression to the following statement was self-paced, and a central crosshair was presented on the screen during the interval between successive statements. Presentation of each new stimulus occurred 500 ms after subject key press response.

The correspondence of the keyboard keys to belief or disbelief was also randomized and the statements themselves were excluded from the MATLAB report to protect subject privacy and as a double-blind measure. The statements were also counterbalanced across each category with the goal of approximately half of the statements yielding a “belief” response. For example, the total number of mathematical statements that were true such as, “2 + 2 = 4,” was equal to the number of mathematical statements that were false. The aim of this is to derive activation related to belief and disbelief in a content-independent manner, with approximately equal numbers of data exemplars in each response category. Each session consisted of~180 trials, which were subsequently used for training and testing of machine learning classifiers.

### Data acquisition and preprocessing

#### fMRI

All structural and functional MRI scans were acquired using a Siemens Allegra 3T scanner (Siemens, Milwaukee, WI). High-resolution structural images were acquired using a magnetization-prepared rapid gradient-echo sequence. Additional scanning parameter details can be found in Harris et al. (2008). Standard preprocessing of data including brain extraction, slice timing correction, motion correction, spatial smoothing using a 5 mm kernel, high-pass filtering, and registration were carried using tools available in FSL (FMRIB Image Analysis Group, http://www.fmrib.ox.ac.uk/fsl) (Woolrich et al., 2001; Jenkinson et al., 2002)

#### EEG

EEG data were recorded using a high density 256-channel GES 300 Geodesic Sensor Net (Electrical Geodesics Inc.) with a sampling rate of 250 Hz in a copper shielded room that was dimly lit. Initial data preprocessing steps were carried out using NetStation 4.4.2 software. These steps included: bandpass filtering from 0.1 to 100 Hz, and a 60 Hz notch filter with a passband gain of –0.1 dB (99%) and stopband gain –40dB (1.0%). We then segmented data 500 ms before and 2500 ms after the stimulus presentation for each event.

Artifact detection for removal of eye movement was accomplished using a moving average of 80 samples with a window size 160 samples to correct for eye movement. Channels that contained >20% error and segments with >10 bad channels were excluded from analysis. Ocular artifact removal was then performed to exclude eye blinks from the analysis, using a blink threshold of 10 μ V/ms for eyeblink detection. Identification and subsequent removal of these artifacts from all channels was accomplished using methods described here (Gratton et al., 1983; Miller et al., 1988). Segments were then averaged across each stimuli condition, and baseline corrected using a 100 ms baseline prior to the stimulus onset for correction.

For our analysis of ERSPs, we bandpass filtered the EEG data into each of the following respective subands: delta (0.1–4 Hz), theta (4–8 Hz), alpha (8–12 Hz), beta (12–20 Hz), and gamma (20–45 Hz). Following preprocessing, the power envelope of each characteristic frequency band was calculated using software developed for this purpose in Matlab (Mathworks, Inc.), and sampled at time points as described below.

### Independent component analysis and feature extraction

#### fMRI

We performed a global ICA computation on each subject's data set. ICA is a powerful tool for finding hidden factors that underlie multivariate data. Known input data, D, is decomposed into a linear combination of statistically independent latent variables, or components, in an unknown mixing system, M. Classic ICA proceeds by the following decomposition:

(1) D=MA.

The matrix A is optimized to obtain statistically independent spatial maps that correspond to various regions of the brain with corresponding temporal aspects. Probabilistic ICA was performed here, using the methodology described above, which forms the basis for the computational program FSL MELODIC, (Beckmann and Smith, 2004).

IC timecourses calculated on training data were sampled at time points corresponding to the maximum predicted BOLD response value. Due to the rapid, self-paced experimental paradigm, multiple belief and disbelief events sometimes occurred within a single repetition time (TR). To avoid overlap in these cases, we included only those data instances whose class label was identical for two or more consecutive trials, effectively reducing the number of exemplars by approximately one third.

In order to extract corresponding IC timecourses from data parsed into the test set for ML purposes, IC spatial masks were binarized and multiplied by the fMRI test data over time. In our previous work, we found that approximately six ICs were effective for classification and describing the data (Anderson et al., 2011). We therefore extracted mean values from each of these IC spatial masks multiplied by the test data, and sampled at time points corresponding to the maximal BOLD activity for each keypad response for subsequent predictive labeling.

#### EEG

Following preprocessing, ICA decomposition was performed on each set of training data for all subjects using the Infomax algorithm (Bell and Sejnowski, 1995) as implemented in the logistic infomax algorithm “binICA” call within EEGLab (Delorme and Makeig, 2004). ICA decomposition of EEG channel data is decomposed into a mixing matrix of weights and IC activations, analogous to equation 1. Segmented data epochs were randomly parsed into ten approximately equal bins. As explained further in the machine learning section, nine of the ten bins were used for training on each cross validation fold. In order to project ICs derived from the training data onto the test data, we calculate the inverse of the mixing matrix and multiply it by the new data as follows:

(2) $${\text{A}}_{\text{Test}}={\text{D}}_{\text{Training}}{\text{M}}_{\text{Training}}^{-1}$$

In each case, our mixing matrix was square and invertible, where the number of ICs was equal to the number of channels. IC data was demeaned for each channel prior to ML analysis. We compared classification of ICs to accuracy using data from each spectral band. In order to accomplish this, we similarly demeaned and squared each spectral time course, and sampled each spectral band along with ICs at time points described below.

***Wavelet Informed EEG IC Sampling.*** In order to determine time points for feature extraction, we utilized time-frequency information contained in the wavelet spectrogram, whose transform is described by equation 3 (Sanei, 2007):

(3) $$W(s,t)=\int x(t)\frac{1}{s}y^{*}\left(\frac{t-t}{s}\right)dt$$

where, ψ is the equation of the mother wavelet, σ is the scaling factor used to dilate and contract the mother wavelet and achieve different pseudofrequencies, τ is the position parameter, and x(t) is the signal being analyzed. Wavelet decomposition was performed on all electrode channels, using the wavelet toolbox within NetStation (v 4.4.2). We used a Morlet wavelet which has a Gaussian shape in both time and frequency domains, with a width of 6 (Tallon-Baudry et al., 1996) and a frequency step of 1 Hz. Power spectrograms in the training data were then averaged across conditions, as has been done by many (e.g., Tallon-Baudry et al., 1997). We baseline corrected and calculated the power by squaring the magnitude of the complex wavelet coefficient and dividing the mean power across the entire segment for each frequency step.

A between-condition difference was then calculated for each channel by averaging the power for each condition at all time points and subtracting the mean power from the opposite condition. The time point that maximized the sum of the power across frequency bands in these differences across all channels was selected for feature extraction as follows:

(4) $$\begin{array}{l} \text{argmax}\left[f_{B},\{t,n\}\right]\;f_{B}=\sum_{jw}\left(E_{B}^{2}-E_{DB}^{2}\right)\\ \text{argmax}\left[f_{DB},\{t,n\}\right]\;f_{DB}=\sum_{jw}\left(E_{DB}^{2}-E_{B}^{2}\right) \end{array}$$

where, *E*_*B*_ is the power for belief at time point *t* in channel *n* averaged across the frequencies contained within the band jω, and E_DB_ is the corresponding measure for disbelief. Feature extraction time points were averaged across all subjects using a leave one out cross validation approach, so wavelet information from the current subject undergoing classification was not used to inform the time point selection. Feature extraction proceeded by sampling power envelopes of either IC time courses or spectral band signatures for each electrode channel at these key discriminatory time points.

### Machine learning classification

Within subject classification of EEG and fMRI data was accomplished using a nested 10-fold cross validation procedure. Events are first parsed randomly into ten bins. Nine bins are then used for training and parameter tuning via an inner cross validation procedure, and the tenth held out data is used as the test set. We tested four machine learning classifiers over a range of complexity: Bayes Net, Support vector machine (SVM) (Burges, 1998; Vapnik, 2000), Adaboost (Viola and Jones, 2001), and J48 decision tree based on the C4.5 decision tree algorithm (Quinlan, 1993). Classification accuracy for each algorithm was assessed via 10-fold cross validation. Hyperparameters were optimized using a 10-fold nested cross validation procedure and ranked features were sequentially added to the training set using a forward feature subset approach, as described in Douglas et al. (2011). For the J48 decision tree, we set the minimum number of instances per leaf to 40. We used implementations of each ML algorithm available in the open source Weka (Waikato Environment for Knowledge Analysis) software.

#### Interpretation of classification

In order to visualize the classification structure for interpretation purposes, we used the WEKA Knowledge Flow tool to illustrate the underlying classifier structure for the J48 decision tree. FMRI IC features that were assigned to either the root node or a decision node further along in the partitioning structure were compared to the GLM data quantitatively by thresholding (*z* ≥ 2.3) and then binarizing each spatial map.

## Results

### EEG belief decision data

**Figure 2:**
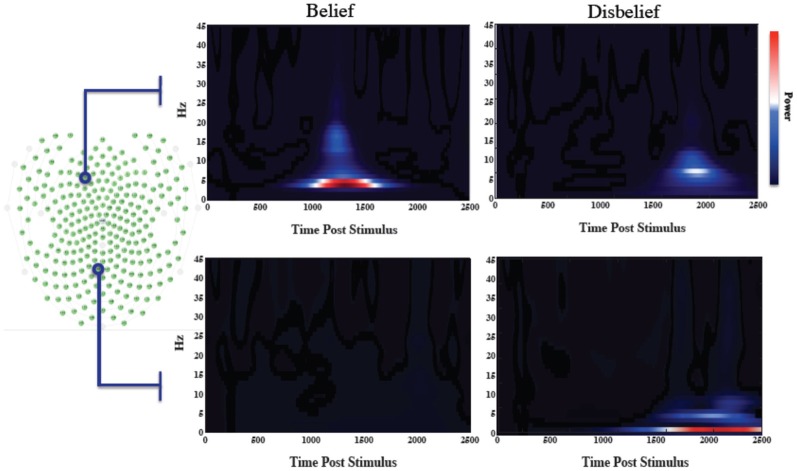
**Wavelet informed sampling of EEG data**. Stimulus-locked wavelet data are shown for a specific individual for illustrative purposes. (Left) Electrode array configuration. (Right) In the top panel, wavelet power data are shown for each category for a particular frontal channel that was used to determine the belief extraction time point. The lower panel similarly shows data from the channel used to determine the disbelief extraction time. Power increases occurred earlier for belief events than for disbelief events.

We averaged the number of responses in each category across subjects to compare the number of data exemplars in each category. Overall, we found that subjects responded “belief” to 45.6% of questions, thus making “disbelief” a slightly more frequent response. The feature extraction time averaged across the group was 578 ± 19 ms earlier than the average time for disbelief. Wavelet spectrograms from an illustrative individual are shown in Figure 2 for channels that mutually maximized belief and disbelief contrasts, along with selected channels location with respect to the electrode channel configuration.

### Classification accuracy interpreting classifier structure

#### Comparing IC spatial maps with GLM results

**Figure 3:**
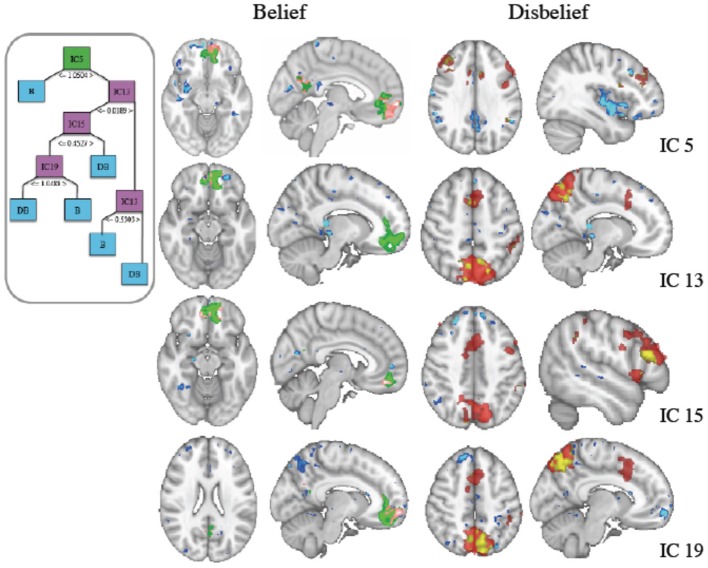
**(Inset) J48 Decision tree structure using independent component (IC) features derived from fMRI data**. The green box at top is the root node, pink boxes are used for subsequent nodes for splitting of the data, and blue boxes indicate terminal decisions or leaf nodes. These IC spatial maps along with general linear model (GLM) contrasts are shown at right. (Left Column) Belief (Green) thresholded z-stat masks generated from a GLM analysis with the contrast of belief-disbelief are overlaid with IC masks (blue) and the voxels that are common to both (pink). (Right Column) Disbelief (red) shown similarly with blue for IC and coregistered voxels in yellow.

Mean 10-fold cross validation accuracy for IC based classification of FMRI was 80.8, 91, 84, and 80% for support vector machine, naïve Bayes, J48 decision tree and k-star classifiers. We generated decision trees for each cross validation fold using reduced error pruning. Based on our nested cross validation, we selected a minimum number of 10 data instances per leaf. The structure of an IC based decision tree classifier using fMRI data from a representative subject's data is shown in Figure 3, with final labels of belief (B) and (DB) indicating the final predicted response.

The root IC and subsequent nodal ICs used in partitioning the data are shown in the inset. IC voxels that also survived threshold in GLM contrasts for belief-disbelief, and disbelief-belief are shown in left and right columns respectively. The root node, IC 5, and ICs 15 and 19 colocalized with belief GLM areas in left middle frontal gyrus and precuneus. ICs 13, 19 and GLM disbelief-belief contrast revealed significant voxels in lateral occipital cortex, whereas paracingulate gyrus was unique to IC 13. Areas that were unique to IC maps included right medial frontal gyrus, right precentral gyrus, right amygdala, and bilateral cingulate cortex.

#### EEG classification accuracy

**Figure 4:**
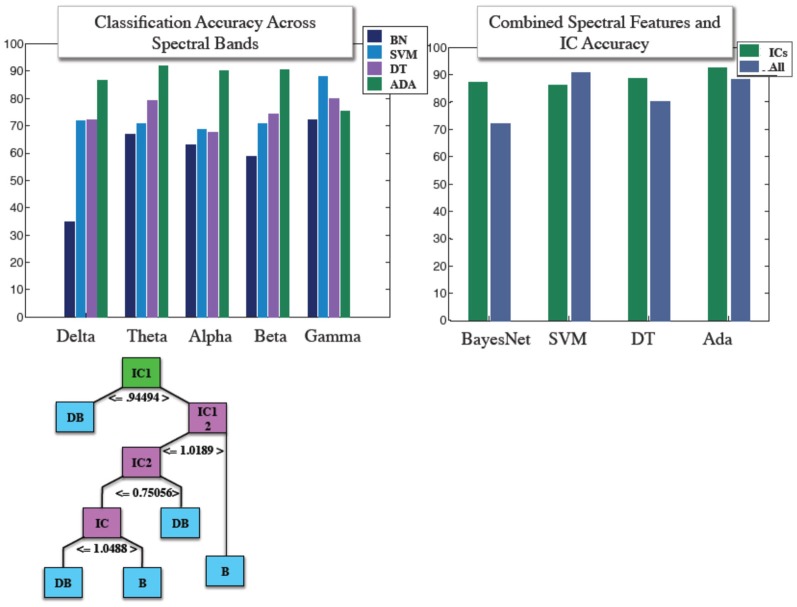
**(Top) Mean classification accuracy achieved using features derived from power envelopes for each spectral band for four classifier algorithms shown in left panel, and IC accuracy with compared with accuracy achieved when combining all spectral features into a single classifier for the same four machine learning algorithms. (Bottom)** Structure of an individual subject example J48 decision tree used in IC based classification.

Similar to our fMRI analysis, we calculated classification accuracy with respect to the four classifiers tested. IC and power envelopes from spectral timecourses were sampled at points determined from wavelets, as described previously. Figure 4 shows the pruned J48 decision tree hierarchical structure for a specific subject's EEG data. Average classification across all four algorithms was 78.8, 77.1, 73.5, 72.2, and 66.1% for gamma, theta, beta, alpha, and delta, respectively. We also stacked all of the spectral features into a “combined” classifier. Mean accuracy for the combined spectral classifier was 82.3%. Overall, the mean of each of these spectral bans as well as the combined spectral classifier were less than 88.6%, the average accuracy achieved using ICs across the four algorithms. Accuracy obtained from using IC features was 85.9, 87.7, 92.6, 88.7%, for support vector machine, BayesNet, AdaBoost, and the J48 Decision tree. Classification accuracy using power envelopes from spectral bands and ICs as features is summarized in Figure 4.

## Discussion

In the current study, we described a method for bivariate classification of belief and disbelief brain states using ICA for both dimension reduction and subsequent feature extraction. We previously developed a method for training a ML classifier on mean time courses extracted from thresholded IC spatial maps (Douglas et al., 2011). In this analysis, we tested the performance of six different ML classifiers in their utility for shattering belief and disbelief data. In this previous analysis, our goal was to develop a classifier that was interpretable by trading off model complexity with error. In this analysis we modeled our classifier output using a sum of exponentials and terminated the addition of more features to the model using the Akaike Information Criterion. While the addition of more features would often diminish the error on the training set marginally, we argued that the sparse classifier would be easier to interpret from a neuroscientific point of view. In the current work, we extended this analysis in two ways. First, we interpreted the classifiers that resulted from our initial analysis by visualizing the extent to which our sparse remaining IC features correlated spatially with the voxel set of significant BOLD activations that resulted from a conventional GLM analysis. Second, we collected EEG data on the same belief decision making paradigm, and applied an analogous ML approach to determine how well IC timecourses could be used to classify EEG belief data.

We first presented the structure of example decision trees classifiers based on fMRI ICs. We found that the spatial patterns of certain IC decision tree nodes were quite similar to conventional GLM results. However, certain IC nodes mapped to regions with unknown relevance to belief decision making. Given the sparsity with which these classifiers were operating, it is possible that these additional areas are involved in some stage of the complex directed processing that occurs in decision making. These regions may only be involved in certain categorical decisions or for certain individuals, and therefore did not survive thresholding in conventional analyses. It is possible that this process may be one mechanism for using IC for exploratory purposes.

We also presented EEG based IC classification results on this same task. The J48 decision tree structure for partitioning data into classes is somewhat similar to decision flow diagrams used for triage in the clinical setting. Decision trees may therefore represent an intuitive structure for interpreting ML features and their output. Overall, we found that ICs proved useful as features for discriminating between the cognitive states of “belief” and “disbelief” at the single trial level in both fMRI and EEG data collected from a high density 256 electrode net. Our IC based classification process can be easily mapped back to the data for interpretive purposes.

### Interpreting diagnostic features: fMRI bold activations and ICs

It is often the case in fMRI decoding studies that the ML process is abstract and can involve thousands of features. While a large vector of features often outperforms a reduced feature set, the margin of improved accuracy may only be slight (e.g., Brodersen et al., 2011; Chu et al., 2012). Selecting relevant features while concurrently minimizing extraneous and redundant features is a key challenges in machine learning (ML) applications, as the performance of certain classifiers degrades with abundant or extraneous information (Kohavi and John, 1997). A parsimonious attribute subset may not only improve the generalization capability of a classifier (Yamashita et al., 2008), but also all for scientific gain when the process is readily intelligible.

Depending on the objective, IC features may be advantageous for ML, as they offer a concise functional representation of the data, which can be easily interpreted and does not require a priori information. It is not surprising that our analysis revealed that certain highly discriminatory ICs coregistered to a large extent with the group level contrasts for Belief-Disbelief and Disbelief-Belief. It is perhaps more interesting to consider regions unique to IC maps.

IC activity not related to the GLM spatial maps that remained in the decision tree after pruning may be meaningful. For example, reading phrases that contain negative emotional associations have been shown to activate amygdala (Osaka et al., 2013), and the amygdala also appears to play a key role in autobiographical memory encoding, consolidation, and retrieval (Markowitsch and Staniloiu, 2011). Others have shown that basolateral amygdala is part of a circuit involved in effort based decision making in rodents (Floresco and Ghods-Sharifi, 2007). Our observation here could therefore relate to emotional or autobiographical memory processing or even the amount of effort required for a particular decision. Activation that did not coregister with GLM regions might also reflect neural activity that non-linearly discriminates between disbelief and belief in a way that t-statistics do not capture. It is also important to note that in our analysis here as well as in (Douglas et al., 2011), we collapsed our analysis across all belief and disbelief categories. Previous work by our group demonstrated that there were no statistically significant differences between these categorical decisions using fMRI GLM analysis (Harris et al., 2008). It is therefore possible that differential regions that we observed, here, reflect categorical differences that did not survive statistical thresholding.

Given the highly complex and distributed nature of the cognitive processing of belief, it is highly likely that the independent multivariate normal assumption of the GLM is violated. However, the nature of this activity is difficult to interpret using simple concepts of up or down regulation of networks. It is also possible that discriminatory information revealed by ICA may not be present in all subjects or all belief/disbelief data exemplars, and therefore a GLM analysis may be underpowered to detect these changes.

### Feature selection and spectral classification in EEG

We used power envelopes derived from spectral bands as features in classification of belief decision-making. Overall, these results demonstrated that the gamma frequency band was most the most discriminatory spectral band for belief/disbelief labeling. Compared to these results, IC features outperformed power envelope in other spectral bands, but was overall similar to the performance of the gamma band features across each of the four classification algorithms discussed here.

A number of studies have found that EEG classification accuracy can vary across frequency subands (e.g., D'Zmura et al., 2009). The functional significance of different neural oscillations are thought to be reflect with different cognitive or neuronal states (Engell et al., 2012). However, interactions across frequencies provide the rich potential for computational encoding of higher order representations. Gamma frequencies, for example, are often modulated by lower frequencies (Buzsáki and Wang, 2012). In terms of decision-making, cross frequency entrainment has been shown to be important in rodent navigation and decision-making (Tort et al., 2008). A number of papers have suggested cross-frequency coupling as a potential mechanism for hierarchical integration of network-level activity (Canolty and Knight, 2010) for higher cognitive processing such as sensory binding. Our wavelet informed feature selection method is consistent with the idea that belief and disbelief would require synchronous activity across frequency bands. However future work is needed to fully understand cross frequency interactions and how they are related to decision processing and whether or not ICs reflect aspects of this coupling.

### fMRI vs. EEG

Overall, IC features derived from EEG data outperformed fMRI data. It is interesting to note that in many of the EEG channels, there were observable event-locked changes in spectral power for both belief and disbelief that were separated in time. Given that these categorical time-frequency changes were separated by ~500 ms, it is unlikely that these temporal changes would be reflected in the BOLD signal. Nonetheless, it is possible that signal changes measured at different times at the same loci on the scalp were actually generated by spatially distinct brain regions, resolvable by fMRI. Future work may involve analyzing EEG in the source domain.

### Decision trees as hierarchical interpretable classifiers

In the present work, we used decision trees for not only classification but also for interpretation of features used in the multistage learning process. Decision trees, which are directed trees with edges and nodes that provide a unique mapping from the root node to a class label. While the overall process is indeed non-linear, decision tree classifiers break down complexity learning problems into the union of a series of simple decisions. Decision trees are perhaps intuitive because provide a quantitative process not all that unlike a decision flow diagrams used commonly in medical triage. When decision tress are used in combination with IC features, decision trees may allow for combining statistical knowledge of activations and deactivations in an interpretable way.

## Conclusions and future work

Overall, these results suggest that ICs may yield an important basis set for classifying complex cognitive processes such as belief and disbelief across both fMRI and EEG neuroimaging modalities. Future work may focus on studying belief and disbelief decision making using concurrently collected EEG-fMRI data. A joint analysis of simultaneous data using methods like joint ICA (e.g., Franco et al., 2008; Edwards et al., 2012) may yield a more in-depth understanding of the neural comparators involved in belief decision-making.

There are strong motivations for understanding the mechanisms underlying veracity assessment, and subsequent decision making about truth content in particular, since a number of potential applications exist given this understanding. When used in combination with machine learning (ML) pattern classification techniques, such knowledge could be used in biomedical applications to drive brain computer interface based communication devices or improved consumer product testing (Calvert and Brammer, 2012). While the ICA computation may be time consuming, in the present work ICs were calculated on training data, and then applied to testing data. Given that the application of ICs to testing data is computationally rapid, real-time application of this methodology may be possible.

### Conflict of interest statement

The authors declare that the research was conducted in the absence of any commercial or financial relationships that could be construed as a potential conflict of interest.
